# Timing of Adjuvant Chemotherapy after Laparotomy for Wilms Tumor and Neuroblastoma

**DOI:** 10.1007/s00383-021-04968-1

**Published:** 2021-07-15

**Authors:** A Ross, O Gomez, X Wang, Z Lu, H Abdelhafeez, AM Davidoff, L Talbot, AJ Murphy

**Affiliations:** a Department of Surgery, St. Jude Children’s Research Hospital, Memphis TN 38105, USA; b College of Medicine, University of Tennessee Health Science Center, Memphis, TN 38105, USA; c Department of Biostatistics, St. Jude Children’s Research Hospital, Memphis TN 38105, USA; d Division of Pediatric Surgery, Department of Surgery, Le Bonheur Children’s Hospital, University of Tennessee Health Science Center, Memphis TN 38105, USA

**Keywords:** Adjuvant chemotherapy timing, chemotherapy initiation, Wilms tumor, neuroblastoma

## Abstract

**Purpose:**

To describe the timing of chemotherapy initiation after surgery for Wilms tumor (WT) and neuroblastoma within a dedicated children’s cancer center.

**Methods:**

A single-institution retrospective cohort study identified patients that underwent resection of unilateral WT or high-risk neuroblastoma and received adjuvant chemotherapy treatment. Adjuvant chemotherapy initiation and postoperative complications were recorded.

**Results:**

Among 47 WT patients, the median time to chemotherapy initiation was 11 days [interquartile range IQR 7-14]. 3 WT patients had post-operative complications, but all preceded chemotherapy. Among 83 patients treated for high-risk neuroblastoma, the median time to chemotherapy was 11 days [IQR 9-14]. High-risk neuroblastoma patients with 30-day postoperative complications had a significantly longer time to initiation of adjuvant chemotherapy (odds ratio 1.13; p=0.008). Many of these complications preceded and delayed the initiation of post-operative chemotherapy. No complications occurred in the group of 12 (25%) WT patients or 16 (19.3%) neuroblastoma patients who started chemotherapy ≤7 days after surgery.

**Conclusion:**

There is no association between early initiation of adjuvant chemotherapy and post-operative complications including wound healing. Early initiation of chemotherapy (≤7 days) is feasible in unilateral WT or high-risk neuroblastoma patients who are otherwise doing well without resulting in a preponderance of wound healing complications.

## Introduction

Over 13,000 new cases of pediatric cancer are diagnosed each year in the United States, with solid tumors representing 30% of these malignancies. Improvements in diagnosis, staging, risk stratification, and treatment strategies have resulted in increased 5-year overall survival rates, reaching roughly 83% currently when compared to 58% 4 decades ago[1]. These improvements have been made possible by integration of multimodality treatment approaches, which may include chemotherapy, radiation, and surgery. Although immunologic or biologic targeted therapies are becoming more common, conventional chemotherapy continues to be one of the main pillars of treatment and consists of antineoplastic agents which preferentially kill rapidly dividing cells via targeting processes that allow for DNA replication or cell division. While the targeting of rapidly dividing cells aims to kill cells with malignant behavior, there is concern that these agents also could impair the healing process after surgery[2].

A common question asked among pediatric surgeons and oncologists is when it is appropriate to start or resume chemotherapy after surgery. After complex or significant tumor resection, there is concern that chemotherapy could impair the healing process and lead to wound complications that would introduce further delays in therapy[2]. There is a paucity of available guidelines to direct decision-making regarding starting chemotherapy after surgery for pediatric cancer patients. For Wilms tumor, protocols suggest initiating chemotherapy no later than 14 days after surgery; however, the earliest timepoint at which it is safe to restart chemotherapy is undefined[3]. The appropriate timing of chemotherapy after surgery for neuroblastoma is less well-defined. With the advent of Enhanced Recovery After Surgery (ERAS) protocols and shorter postoperative hospitalizations in pediatric surgical patients, many patients could become eligible for earlier initiation of adjuvant chemotherapy[4, 5]. Key aspects of ERAS management being actively studied in these patient groups include extensive parental preoperative briefing on postoperative expectations, preoperative oral hydration, opioid reduction by placement of a pre-anesthetic epidural catheter or nerve block, minimization of intraoperative fluid and opioid administration, discontinuation of the nasogastric tube at extubation, removal of the urinary catheter on postoperative day (POD) 1, multimodal postoperative pain control including Tylenol and Toradol, early ambulation, and advancement of the postoperative diet as tolerated [4, 6].

Neuroblastoma and Wilms tumor are the most common intraabdominal tumors of childhood and require major surgical resection in most cases [7, 8]. Induction chemotherapy for high-risk neuroblastoma typically consists of 5 cycles of chemotherapy, with surgery following cycle 4 or 5. Commonly used chemotherapeutic agents during induction therapy for high-risk neuroblastoma include cyclophosphamide, topotecan, cisplatin, vincristine, doxorubicin, and etoposide. In addition, patients treated on our institutional NB2012 protocol also received anti-GD2 antibody treatment during induction therapy [11, 12]. In contrast, surgery typically precedes chemotherapy for unilateral Wilms tumor patients. Chemotherapy for Wilms tumor typically consists of vincristine and actinomycin-D with or without doxorubicin depending on the stage of disease. High-risk Wilms tumor may also warrant intensification of the chemotherapy regimen with additional agents including etoposide, cyclophosphamide, and carboplatin. Many of these chemotherapeutic agents have well known significant adverse effects due to mechanisms of action including inhibition of cell metabolism, division, and angiogenesis [13]. Specifically, cyclophosphamide, doxorubicin, and cisplatin may complicate post-operative recovery by various mechanisms including myelosuppression, decreased collagen synthesis, decreased proliferative phase of wound healing [14–16]. The aim of this retrospective research study is to better understand and describe the timing of adjuvant chemotherapy initiation after surgery for Wilms tumor and neuroblastoma within a dedicated children’s cancer center. An exploratory aim of this study is to determine if there is any association between the timing of adjuvant chemotherapy initiation and postoperative complications including wound healing.

## Materials and Methods

This study was declared exempt and deemed to be non-human subjects research by the St. Jude Children’s Research Hospital Institutional Review Board; waiver of informed consent was obtained. Our institutional surgical database was queried for patients less than 19 years of age with a diagnosis of Wilms tumor or neuroblastoma who underwent exploratory laparotomy with major surgical resection (radical nephroureterectomy with lymph node sampling for Wilms tumor or adrenal/retroperitoneal mass resection with or without lymphadenectomy for neuroblastoma) between 2008 and 2019. We included patients with unilateral Wilms tumor or high-risk neuroblastoma in our analysis to focus on two groups of patients with relatively standardized treatment approaches. Patients who received adjuvant chemotherapy at other institutions were excluded from analysis to ensure accurate assessment of adjuvant chemotherapy initiation timing. Patients with bilateral Wilms tumor, and low- or intermediate-risk neuroblastoma were excluded from the analysis because of variations in the therapeutic approach to these patient populations. Data abstracted from the electronic medical record included biological sex, race, patient age at diagnosis, disease stage (Children’s Oncology Group stage for Wilms tumor and International Neuroblastoma Staging System and International Neuroblastoma Research Group Staging System for neuroblastoma), N-Myc amplification status for neuroblastoma, operation performed, adjuvant chemotherapy drugs and protocol, intraoperative complications, 30-day postoperative complications classified according to Clavien-Dindo, and time to adjuvant chemotherapy initiation[8, 17–19]. Instances of documented clinical decisions to delay initiation of adjuvant chemotherapy were recorded and the reason for the decision was noted.

Descriptive statistics were calculated including mean with standard deviation and median with interquartile range for continuous variables and percentage of total for categorical variables. Time to adjuvant chemotherapy initiation was compared between patients with and without postoperative complications using the Wilcoxon rank-sum test and by calculating the odds ratio. Cumulative incidence curves were calculated showing the time to initiation of adjuvant chemotherapy for patients with and without postoperative complications. A two-sided p value of <0.05 was considered statistically significant for all tests. Statistical analysis was performed in the R software package (version 3.6.3, R Core Team, Vienna, Austria).

## Results

### Unilateral Wilms tumor

#### Patient characteristics

**Fig 1:**
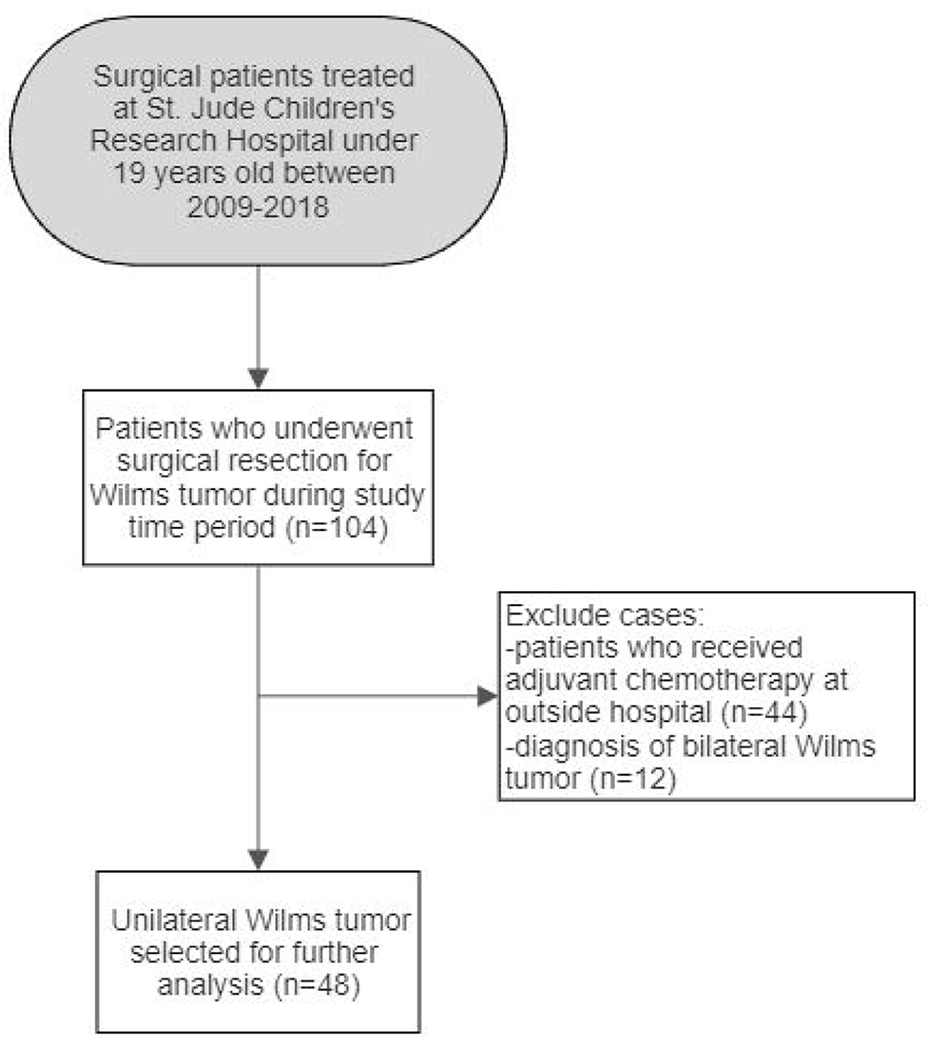
Selection of Wilms tumor study cohort. 48 patients were selected for further analysis according to inclusion criteria

A total of 104 patients who underwent surgical resection for Wilms tumor were identified during the study period. 48 patients with unilateral Wilms tumor who received adjuvant chemotherapy at our institution were selected for further analysis (Figure 1). All Wilms tumor patients were treated on Children’s Oncology Group (COG) studies or “as per” in the absence of open protocols. Among those treated for unilateral Wilms tumor, median time to adjuvant chemotherapy initiation was 11 days (interquartile range [IQR 7-14]). 12 patients (25%) initiated adjuvant chemotherapy in ≤7 days. Among this group of patients who started adjuvant chemotherapy in ≤7 days after surgery, 1 patient started in 4 days, 1 patient started in 5 days, 4 patients started in 6 days, and 6 patients started in 7 days. The characteristics of unilateral Wilms tumor patients including demographics, disease staging, adjuvant chemotherapy regimen, and frequency of post-operative complications are shown in Table 1.

#### Post-operative complications in unilateral Wilms tumor patients

Patients being treated for unilateral Wilms tumor with adjuvant chemotherapy were assessed for post-operative complications in relation to initiation of chemotherapy using the Clavien-Dindo classification system. Of the 48 patients in this cohort, 3 patients had a post-operative complication. 2 patients had intussusception requiring reoperation on post-operative day (POD) 3 and 4. One patient had an adhesive small bowel obstruction requiring reoperation with adhesiolysis on POD6. All three complications were classified as Clavien-Dindo class 3B. All complications occurred before the initiation of adjuvant chemotherapy, and in all three instances led to delayed initiation of adjuvant chemotherapy. No patient experienced a wound complication after surgery for unilateral Wilms tumor in this series and thus no association could be made between timing of adjuvant chemotherapy initiation and wound complications. No post-operative complications occurred in the group of 12 patients who started adjuvant chemotherapy ≤7 days after surgery.

#### Factors that delay initiation of adjuvant chemotherapy in unilateral Wilms tumor patients

Initiation of adjuvant chemotherapy was delayed in a total of 5 Wilms tumor patients. In patients being treated for unilateral Wilms tumor, median time to initiation of chemotherapy was 11 days [IQR 7, 14]. To understand the factors that influence timing of adjuvant chemotherapy, descriptive data were collected. In the unilateral Wilms tumor group, recovery from a 2^nd^ exploratory laparotomy (n=3, delayed to 12 (2x) and 21 days), video assisted thoracoscopic surgery (VATS) (n=1, 21 days), and staged thoracotomy (n=1, 35 days) were the only factors that led to a clinical decision to delay adjuvant chemotherapy.

#### Timing of adjuvant chemotherapy and post-operative complications in unilateral Wilms tumor

**Fig 2:**
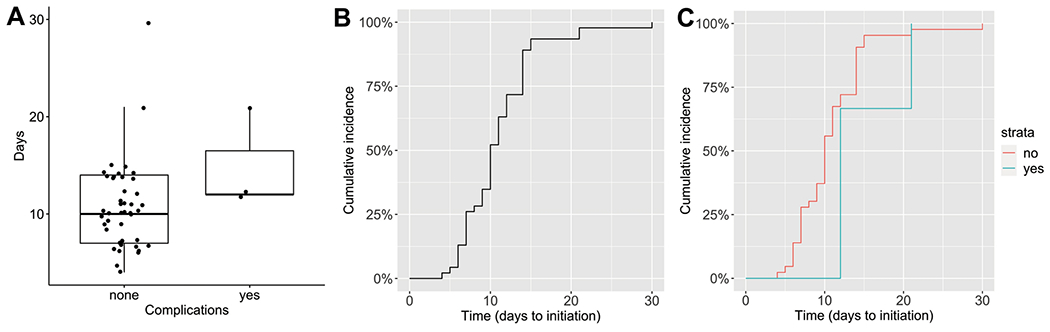
Postoperative complications and timing of adjuvant chemotherapy initiation in unilateral Wilms tumor patients. (A) Boxplot representation of days to initiation of adjuvant chemotherapy in unilateral Wilms tumor patients without and with post-operative complications. Boxplots are drawn with the dark line representing the median and edges of box representing the interquartile range. (B) Cumulative incidence curve demonstrating the time to initiation of adjuvant chemotherapy in all unilateral Wilms tumor patients in the cohort. (C) Cumulative incidence curves comparing the initiation of adjuvant chemotherapy between patients without (red) and with (blue) post-operative complications

To explore the relationship between timing of adjuvant chemotherapy initiation and complications occurring within 30 days of surgery for unilateral Wilms tumor, the time to initiation of adjuvant therapy was compared between patients with and without 30-day postoperative complications. The 45 (93.8%) patients without a post-operative complication had a median time to initiation of chemotherapy of 10[IQR 7-14] days and the 3 patients (6.2%) with a post-operative complication had a median time to initiation of 12 days ([IQR 12-16.5]; Wilcoxon rank sum test p=0.102); Figure 2. Therefore, no significant differences were detected in timing to adjuvant chemotherapy initiation between patients with and without post-operative complications; however, the number of post-operative complications in this cohort is very small and is likely underpowered to detect a difference with this small effect size. Cumulative incidence curves were developed to display the initiation of adjuvant chemotherapy in unilateral Wilms tumor patients with and without post-operative complications (Figure 2).

### High-Risk Neuroblastoma

#### Patient Characteristics

**Fig 3:**
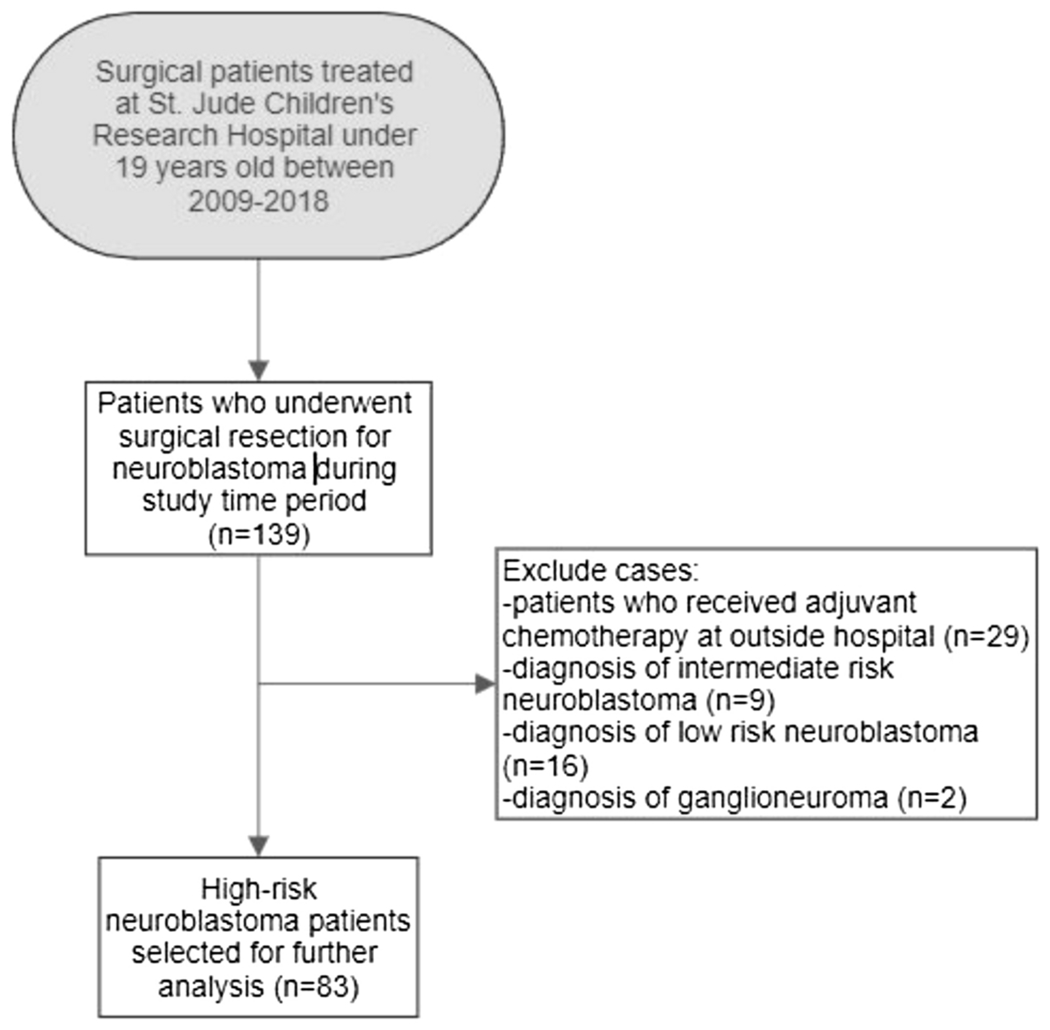
Selection of high-risk neuroblastoma study cohort. 83 patients were selected for further analysis according to inclusion criteria

A total of 139 patients treated at St. Jude Children’s Research Hospital for neuroblastoma with both surgery and adjuvant chemotherapy were identified. Of the original cohort, 83 patients with high-risk neuroblastoma were selected for further analysis due to greater standardization of the neoadjuvant and adjuvant treatment protocols (Figure 3). The majority of patients were treated on one of three IRB-approved institutional protocols for high-risk neuroblastoma: NB91 (14%), NB2008 (1%), or NB2012 (45%) (Table 2). In the high-risk neuroblastoma group, the median time to initiation of adjuvant chemotherapy was 11 days [IQR 9-14], 16 patients (19.3%) started chemotherapy in ≤7 days after surgery. Among these 16 patients, 3 patients started chemotherapy in 4 days, 6 patients in 6 days, and 7 patients in 7 days. The characteristics of the high-risk neuroblastoma cohort include demographics, disease staging, adjuvant multidrug chemotherapy regimen, and common post-operative complications and are shown in Table 2.

#### Post-operative complications in high-risk neuroblastoma patients

Descriptive data identifying post-operative complications in high risk-neuroblastoma patients was collected to assess any relationship to initiation of chemotherapy. In the high-risk neuroblastoma cohort, 14 (16.9%) of the 83 patients had a post-operative complication. The following complications were observed: chylous ascites (n=4), pleural effusion (n=2), surgical wound infection or dehiscence (n=6), and other (n=2). Of these complications, 7 occurred prior to initiation of adjuvant chemotherapy (3 with chylous ascites, 2 with pleural effusion, 2 with wound infection) and 7 occurred after (1 with chylous ascites, 4 with wound dehiscence, and 2 with other complications, including inability to bear weight following surgery and subglottic stenosis secondary to endotracheal intubation). None of the 7 complications occurring after adjuvant chemotherapy initiation led to a clinical decision to delay additional treatment. All complications were classified according to the Clavien-Dindo method; 6 complications were classified as class 1, 5 as class 2, 1 as class 3A, 1 as class 3B, and 1 as class 4B. No post-operative complications occurred in the group of 16 patients who started adjuvant chemotherapy in ≤7 days after surgery.

#### Factors that delay initiation of adjuvant chemotherapy in high-risk neuroblastoma patients

Initiation of adjuvant chemotherapy was delayed in a total of 13 high-risk neuroblastoma patients. The documented factors that led to a clinical decision to delay adjuvant chemotherapy included: significant intra-operative blood loss with prolonged post-operative recovery (postoperative ileus greater than one week, significant pain control issues greater than one week, postoperative hospitalization greater than two weeks, or severe deconditioning due to postoperative critical illness and ICU stay; n=5), post-operative chylous ascites (n=1), pleural effusion (n=1), resistant hypertension (n=2), wound infection (n=2), and patient preference/social situation (n=2). Delays of >20 days occurred in 7 patients for the following reasons: significant intra-operative blood loss (n=2), acute kidney injury (n=1), parent preference (n=1), central line infection (n=1), venous thromboembolism (n=1), and additional stem cell harvest (n=1).

#### Timing of adjuvant chemotherapy and post-operative complications in high-risk neuroblastoma

**Fig 4:**
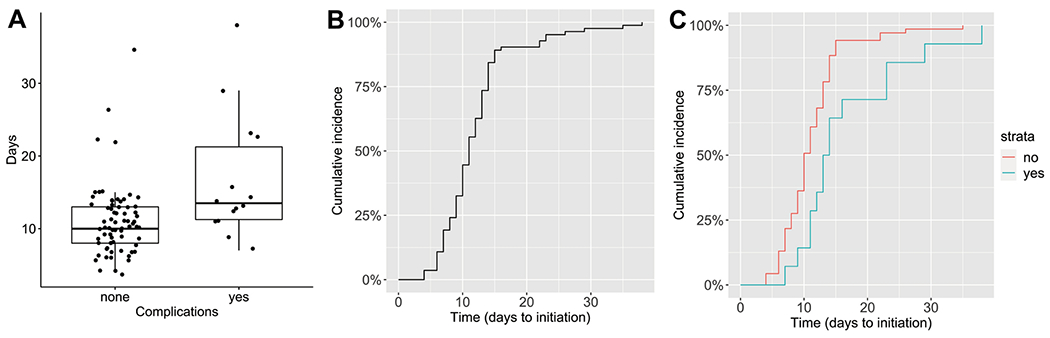
Postoperative complications and timing of adjuvant chemotherapy initiation in high-risk neuroblastoma patients. (A) Boxplot representation of days to initiation of adjuvant chemotherapy in high-risk neuroblastoma patients without and with post-operative complications. Boxplots with dark line representing median and edges of box representing the interquartile range. (B) Cumulative incidence curve demonstrating the time to initiation of adjuvant chemotherapy in all high-risk neuroblastoma patients in the cohort. (C) Cumulative incidence curves comparing the initiation of adjuvant chemotherapy between patients without (red) and with (blue) post-operative complications.

To explore the relationship between timing of adjuvant chemotherapy initiation and complications occurring within 30 days of surgery for high-risk neuroblastoma, the time to initiation of adjuvant therapy was compared between patients with and without 30-day postoperative complications. The 69 (83.1%) patients without a post-operative complication had a median time to initiation of chemotherapy of 10 [IQR 8-13] days and the 14 patients (16.9%) with a post-operative complication had a median time to initiation of 13.5 days ([IQR 11.3-21.3]; Wilcoxon rank sum test p=0.006); Figure 2. Therefore, high-risk neuroblastoma patients with 30-day postoperative complications were found to have a significantly longer time to initiation of adjuvant chemotherapy (odds ratio 1.13; p=0.008). Cumulative incidence curves were developed to display the initiation of adjuvant chemotherapy in high-risk neuroblastoma patients with and without post-operative complications (Figure 4).

## Discussion

This retrospective study describes the timing of adjuvant chemotherapy initiation in patients who underwent surgery for unilateral Wilms tumor or high-risk neuroblastoma at a dedicated children’s cancer center. These two specific groups were selected for analysis due to the standardization of their treatment regimens, as opposed to patients with bilateral Wilms tumor who are treated with uniform neoadjuvant chemotherapy or low or intermediate risk neuroblastoma, who may receive no chemotherapy or only neoadjuvant chemotherapy. For both groups, the median time to initiation of adjuvant therapy was between 1 to 2 weeks after surgery. No postoperative complications occurred in the group of 12 (25%) unilateral Wilms tumor patients or the group of 16 (19.3%) high-risk neuroblastoma patients who started chemotherapy in ≤7 days after surgery, suggesting that early initiation of chemotherapy is safe in appropriately selected patients. These data will be helpful for pediatric surgeons and oncologists debating when to start chemotherapy in patients who are otherwise doing well after major cancer operations.

For unilateral Wilms tumor, there were no wound or healing complications in the cohort and all postoperative complications preceded the initiation of adjuvant chemotherapy. Protocol guidelines call for initiation of chemotherapy between 7-14 days after surgery. Delays in adjuvant radiotherapy for Wilms tumor beyond 10-14 days after nephrectomy have been associated with increased local recurrence[3]. There was no significant association between post-operative complications and adjuvant chemotherapy initiation in this cohort, likely due to the small number of events. While the study is underpowered to truly determine the association between uncommon post-operative complications and chemotherapy initiation, the take home message is that wound complications are exceedingly rare after laparotomy for Wilms tumor and the fear of wound complications should therefore not influence the decision to initiate adjuvant chemotherapy in a timely fashion. Our data support the protocol recommendation that adjuvant chemotherapy be started within two weeks of surgery for Wilms tumor and may suggest that even earlier initiation of adjuvant chemotherapy is feasible and safe. Furthermore, our data show that initiation of adjuvant chemotherapy within one week of surgery is feasible in selected postoperative unilateral Wilms tumor patients who are otherwise doing well. Enhanced Recovery after Surgery (ERAS) protocols are currently being studied in pediatric oncology patients and may increase the number of unilateral Wilms tumor patients or high risk-neuroblastoma patients in whom initiation of chemotherapy within one week of surgery is feasible. 25% of patients with unilateral Wilms tumor in our study were able to safely initiate adjuvant chemotherapy in less than or equal to 7 days after surgery. None of these patients had post-operative complications, suggesting that early initiation of chemotherapy (≤7 days) is feasible and safe.

The current COG high-risk neuroblastoma protocol ANBL1531 recommends that surgery avoid delays of more than six weeks between chemotherapy cycles; however, more specific descriptions of adjuvant chemotherapy initiation are lacking in the surgical literature. For high-risk neuroblastoma, this study shows a statistically significant association between post-operative complications and longer time to initiation of adjuvant chemotherapy. The question then becomes: are post-operative complications delaying chemotherapy or is chemotherapy resulting in post-operative complications? This retrospective, non-randomized study cannot definitively answer this question. However, to guide our interpretation of the current results, we reviewed the complications to determine whether they occurred before or after adjuvant chemotherapy initiation. One half of complications occurred prior to the initiation of adjuvant chemotherapy and one half after. No complications occurring after restarting chemotherapy led to a clinical decision to suspend or delay further chemotherapy treatment. Clinical decision to delay chemotherapy was attributed to intraoperative complexity or complications (high blood loss surgery) or complications that manifested in the postoperative period in most cases. In terms of wound complications, these were relatively rare with half manifesting before the initiation of chemotherapy and half after. Therefore, we do not feel that fear of wound complications should influence the decision to initiate adjuvant chemotherapy in patients with high-risk neuroblastoma unless a complication has already begun to manifest at the time the decision is being made. 19.3% of the patients in our study with high-risk neuroblastoma were able to safely initiate adjuvant chemotherapy in less than or equal to 7 days after surgery and none of these patients had a post-operative complication, suggesting that early initiation of chemotherapy is feasible, safe, and not associated with wound complications. Based on these data, we are now open to initiation of chemotherapy at our institution based on the patient’s recovery (pain controlled on oral medications, ambulatory, voiding, return of bowel function, tolerating diet) and do not adhere to any specific timeline or delay before initiating chemotherapy.

This study has some inherent limitations, which include its retrospective, single-institution nature. Delays in chemotherapy were defined as a documented clinical decision to postpone initiation of adjuvant chemotherapy, but this retrospective approach could be subject to inconsistent documentation or documentation bias. Due to low overall incidence of post-operative complications in the unilateral Wilms tumor cohort, this study is likely underpowered to detect differences in the timing of adjuvant chemotherapy in this group. A randomized study to determine whether early initiation of chemotherapy (less than or equal to 7 days) influences postoperative wound complications is unlikely to be feasible given the low incidence of wound complications and the influence of a variable postoperative recovery course on the readiness for chemotherapy initiation in individual patients. High-risk neuroblastoma patients uniformly receive multi-agent induction chemotherapy prior to surgery, which can affect their nutritional and immunologic status and could influence the risk of post-operative complications including wound healing as much or more than the timing of adjuvant chemotherapy initiation.

When resuming chemotherapy after tumor resection, oncologists prefer to minimize the time elapsed between cycles. However, the decision to resume chemotherapy is generally left to the discretion of the surgeon because of the perceived risks of post-operative complications including wound infection and delayed wound healing. Given the scarcity of previous studies and formal guidelines in the pediatric population, surgeons must use their best judgement and assessment of the post-surgical course to guide the timeline to resumption of chemotherapy. However, the results of our single-institution study show that the risk of post-operative complications associated with resumption of chemotherapy is low, and 25% of Wilms tumor and 19% of high-risk neuroblastoma patients were able to safely initiate chemotherapy within 7 days of surgery without any postoperative complications. In a previously published study by Imran et al. (2009), investigation of initiation of adjuvant chemotherapy in osteosarcoma patients reached a similar conclusion, finding no significant association between early initiation of adjuvant chemotherapy and post-operative complications or event-free survival [20]. Based on this data, we suggest that surgeons weigh the risks of post-operative complications, which may prolong hospital stay or decrease survival, versus the benefits of early chemotherapy initiation in their decision making. Patients who may be candidates for initiation of adjuvant chemotherapy within 7 days of surgery will meet clinical criteria including uncomplicated intraoperative course, early return of bowel function, adequate pain control, and absence of early postoperative complications. Pediatric surgeons and oncologists should collaborate to ensure that patients return to clinic to resume chemotherapy as soon as is prudent given their post-surgical course and disease status. St. Jude Children’s Research Hospital is an institution uniquely focused on treating pediatric cancer. We believe that our focus and experience have enabled earlier initiation of adjuvant chemotherapy in some cases of unilateral Wilms tumor and neuroblastoma, however our approach is consistent with standard postoperative management and decision-making and can be replicated at any institution caring for pediatric oncology patients. The results of the current study can be used as a reference for care and should be investigated prospectively in future studies to better establish guidelines regarding how quickly chemotherapy can be safely initiated following tumor resection, and we are currently actively participating in an ongoing prospective, multi-institutional ERAS protocol for pediatric oncology patients that will record initiation of chemotherapy in relation to perioperative care regimen.

## Figures and Tables

**Table 1.** **Unilateral Wilms tumor** patient demographics and clinical characteristics. Numbers are represented as number (%) or median [interquartile range]. VA – vincristine and actinomycin-D, VAD – vincristine, actinomycin-D, doxorubicin

	Total N = 48
**Age (years) [IQR]**	3.5 [4.00]
**Biological sex**	
Female	28 (58.3)
Male	20 (41.7)
**Race**	
White	28 (58.3)
Black	11 (22.9)
Asian	1 (2.0)
Hispanic	1 (2.0)
Other	4 (8.3)
**Disease Stage**	
I	5 (10.4)
II	12 (25.0)
III	12 (25.0)
IV	19 (39.6)
**Adjuvant Chemotherapy Drugs**	
VA	15 (31.3)
VAD	27 (56.3)
Other	6 (12.5)
**30-day postoperative complications**	3 (6.3)
Intussusception	2 (4.2)
Small Bowel Obstruction	1 (2.1)
Wound dehiscence or infection	0 (0)
**Adjuvant Chemotherapy Initiation**	
Time to Initiation (Days) [IQR]	11.0 [7, 14]
≤7 days	12 (25)
8-14 days	29 (60.4)
≥15 days	7 (14.6)

**Table 2.** **High-risk neuroblastoma** patient demographics and clinical characteristics. Numbers are represented as number (%) or median [interquartile range].

	Total N = 83
**Age (years) [IQR]**	3.0 [2.5]
**Biological Sex**	
Female	45 (54.2)
Male	38 (45.8)
**Race**	
White	57 (68.6)
Black	23 (26.4)
Other	3 (3.6)
**Disease Stage (INSS)**	
II	1 (1.2)
III	10 (12.0)
IV	72 (86.8)
**Disease Stage (INRG)**	
L1	1 (1.2)
L2	9 (10.8)
M	72 (86.8)
**NMYC amplification**	25 (30.1)
**Adjuvant Chemotherapy Protocol**	
NB91	12 (14.4)
NB2008	1 (1.2)
NB2012	45 (54.2)
Other	25 (30.2)
**30-day postoperative complications**	
Chylous ascites	4 (4.8)
Pleural effusion	2 (2.4)
Wound dehiscence or infection	6 (7.2)
Other	2 (2.4)
**Adjuvant Chemotherapy Initiation**	
Time to Initiation (days) [IQR]	11.0 [9, 14]
≤7 days	16 (19.3)
8-14 days	54 (65.1)
≥15 davs	13 (15.6)
